# Manipulation of local optical properties and structures in molybdenum-disulfide monolayers using electric field-assisted near-field techniques

**DOI:** 10.1038/srep46004

**Published:** 2017-04-05

**Authors:** Junji Nozaki, Musashi Fukumura, Takaaki Aoki, Yutaka Maniwa, Yohei Yomogida, Kazuhiro Yanagi

**Affiliations:** 1 Department of Physics, Tokyo Metropolitan University, 1-1 Minami-Osawa, Hachioji, Tokyo 192-0397, Japan

## Abstract

Remarkable optical properties, such as quantum light emission and large optical nonlinearity, have been observed in peculiar local sites of transition metal dichalcogenide monolayers, and the ability to tune such properties is of great importance for their optoelectronic applications. For that purpose, it is crucial to elucidate and tune their local optical properties simultaneously. Here, we develop an electric field-assisted near-field technique. Using this technique we can clarify and tune the local optical properties simultaneously with a spatial resolution of approximately 100 nm due to the electric field from the cantilever. The photoluminescence at local sites in molybdenum-disulfide (MoS_2_) monolayers is reversibly modulated, and the inhomogeneity of the charge neutral points and quantum yields is suggested. We successfully etch MoS_2_ crystals and fabricate nanoribbons using near-field techniques in combination with an electric field. This study creates a way to tune the local optical properties and to freely design the structural shapes of atomic monolayers using near-field optics.

Recently, monolayer transition metal dichalcogenides (TMDCs) have attracted much interest as nanomaterials for next generation optoelectronic devices because of their large nonlinear optical processes^1^, single photon emissions^2^, and interlayer excitonic transitions^3^, etc. Such remarkable optical processes have been observed in peculiar local sites, such as grain boundaries, defects, and hetero stacked parts^1^,^2^,^3^,^4^,^5^. Such optical properties are strongly modulated by local carrier densities, electric fields, and local structures^6^,^7^,^8^,^9^. For example, the optical properties of TMDCs can be modulated by the application of an electric field through carrier injections^6^,^7^ and Stark effects^9^. It is highly desirable to develop a method to clarify the local optical properties in high spatial resolution and, at the same time, precisely tune those properties for their optoelectronic applications. For that purpose, we combined scanning near-field optical microscopy (SNOM) techniques and electric field applications.

SNOM is a technique that can directly reveal local optical characteristics, such as photoluminescence (PL)^10^,^11^,^12^, and the optical absorption spectra^13^,^14^ of 2D materials with a spatial resolution of approximately 100 nm^15^,^16^. However, these previous SNOM results were measured without any perturbations in the samples from an electric field. As a result, using conventional SNOM approaches we cannot manipulate the local optical properties. To overcome this issue, we created a setup to apply DC bias voltages from the cantilever of a SNOM and tried to reveal the local optical properties while simultaneously manipulating the properties using the electric field from the cantilever. As described below, the local PL spectra on a site of a MoS_2_ monolayer was reversibly modulated with a spatial resolution of approximately 100 nm, and the evaluation of its optical properties was achieved by this method. In addition, in the negative bias voltage region, we found that the structure of the MoS_2_ monolayer was unstable and sometimes decomposed. On the basis of this observation, MoS_2_ crystals could be etched by near-field light in combination with negatively biased voltages. Furthermore, using this technique, we fabricated MoS_2_ nanoribbons with widths of almost 500 nm from triangular MoS_2_ monolayer crystals.

## Results

### Local carrier injection control and its site dependence in MoS_2_ monolayers

**Figure 1:**
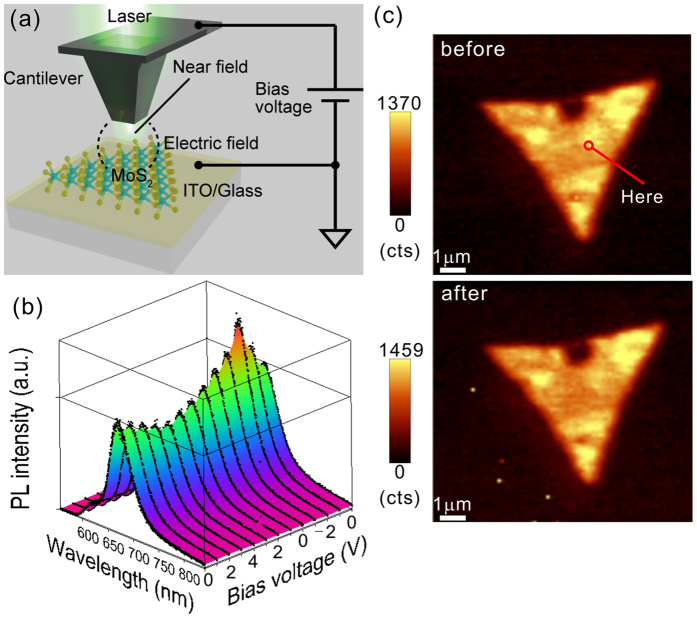
PL modulation in the MoS_2_ monolayer by local carrier injection control using the electric field-assisted scanning near-field optical microscopy system. (**a**) A schematic illustration of the experimental setup of our electric field-effect scanning near-field optical microscopy system. (**b**) Modulation of the local PL spectra on the CVD-grown MoS_2_ monolayer at the selected point shown in (**c**) by systematically shifting the bias voltages. The bias voltage was sequentially changed from 0 V to 4 V, then to −2 V and returned to 0 V. (**c**) Local PL mappings image before and after the bias voltage modulations described in (**b**).

To investigate the local optoelectronic response of MoS_2_ monolayers in the presence of an electric field, we made a setup to introduce a DC bias voltage between the SNOM cantilever and sample. Figure 1a shows a schematic illustration of our experimental setup for the electric field-effect SNOM system. The SNOM cantilever is made of SiO_2_ coated with aluminum, and it has a small aperture with a 60 nm diameter. The excitation laser (532 nm CW laser) was introduced to the aperture of the cantilever, and near-field lights were produced from the nanoscale aperture. The optical characteristics at a site were evaluated using this near-field technique. At the same time, DC electric fields were applied on the same site by the bias voltages from the top of the cantilever, and the PL from the samples was detected from an inverted lens underneath the sample. To apply bias voltages between the sample and the cantilever, MoS_2_ monolayers, which were synthesized by a chemical vapor deposition (CVD) method, were transferred onto conductive indium tin oxide (ITO) substrates. All measurements were performed in a N_2_ flow atmosphere at room temperature. (See Methods and Supplementary Fig. S1 for the detailed experimental setup and sample preparation methods.)

Figure 1b shows the local PL spectra modulated by applying a bias voltage on a selected local site of a MoS_2_ monolayer crystal. It is clearly seen that the PL peak intensities were systematically modulated by the change of the bias voltage. As the bias voltage changed to a positive direction, the PL peak intensities decreased and were minimized at 4 V. This indicates that local electron injections occurred at the site due to the electric field application^7^. In contrast, when the bias voltage changed to a negative direction, the PL intensities increased drastically and were maximized at −2 V, indicating dedoping of the negatively charged state of the site. Those observations are similar to the electric field-effect on the PL of MoS_2_ monolayers by back-gating approaches^7^. This PL intensity modulation was reversible and repeatable within this voltage range (See Supplementary Fig. S2), and the sample was not damaged, as shown in Fig. 1c. Figure 1c shows SNOM PL mapping images before and after the PL modulation. As shown in these two images, there was no damage to the crystal structure due to PL modulation control. These results suggest that the local reversible carrier injection on MoS_2_ in the range of a few tens of nanometers was achieved by our technique. In addition, slight peak shift of PL depending on the bias voltage was also observed (Supplementary Fig. S3). Such peak shift would be caused by the increase of trion by electron injections as reported by ref. 7, supporting carrier injections by our approaches. The spatial resolution of our SNOM system can be estimated approximately 100 nm from the obtained near-field PL mapping image (See Supplementary Fig. S4). We assume that the presence of the aluminum oxide layers, which were naturally formed on the surface of the cantilever, enabled the application of the electric field to the sample during contact mode measurements using SNOM. Compared to the previous results^7^, the thickness of the aluminum oxide layer and the injected carrier density were estimated to be nearly 30 nm and 10^13^ cm^−2^, respectively.

To clarify the doping effect in TMDCs by bias voltages in more detail, Raman spectroscopy would give us additional information^17^,^18^. However, in the case of aperture-type SNOM techniques, as used in this study, the light intensity, 10 nW μm^−2^, is too low to detect a Raman signal. The detection of the Raman signal with the aperture-type SNOM method remains as one of technical challenges in the field. Thus, we just focus on the PL characteristics in this study.

**Figure 2:**
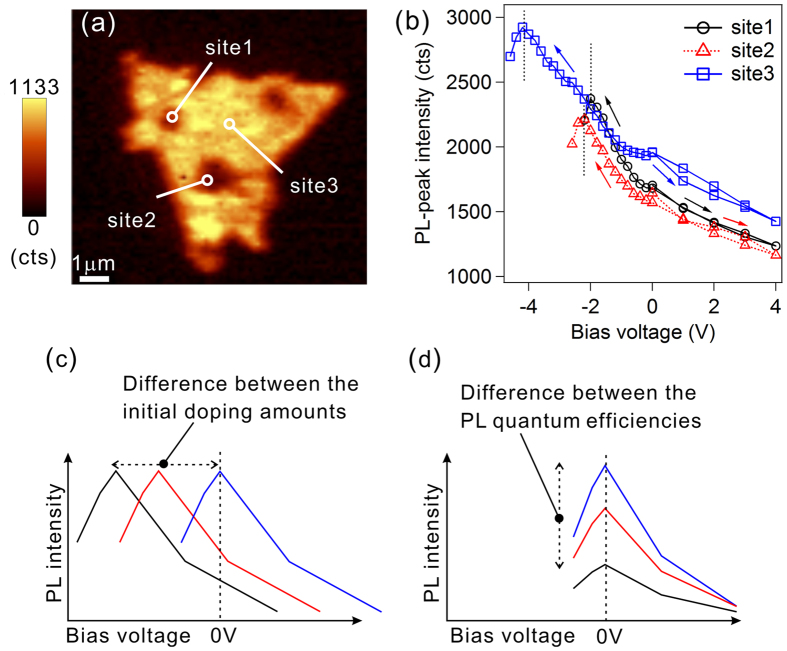
Relationships between the bias voltages and local PL peak intensities at several sites of the MoS_2_ monolayer. (**a**) A SNOM PL mapping image of a MoS_2_ monolayer whose PL intensity indicated local site dependence. (**b**) Bias voltage dependence of the local PL peak intensities at each of the local sites shown in the panel (**a**). Bias voltages were sequentially changed according to the directions indicated by the arrows from 0 V to the positive direction and to the negative direction. (**c**) A schematic illustration showing the influence of different initial doping amounts and (**d**) a schematic illustration showing the influence of different PL quantum efficiencies on the PL intensity and bias voltages.

As shown in Fig. 1c, the PL map was not homogeneous, but there were bright and dark PL sites at a 0 V bias voltage. If the differences between the PL intensities reflected the initial n-doping levels due to the presence of defects or dopants^19^,^20^,^21^, then the charge neutral points should be different depending on the local sites. Such points would be identified by our technique by evaluating the bias voltages that induces the maximum PL intensities. To clarify this points, we investigated the relationships between the PL intensities and bias voltages at different sites. We investigated three sites: one was bright, and the other two were dark at 0 V (Fig. 2a). There were voltage points, which are termed as the saturation voltages, that saturated the PL peak intensities at negative bias voltages at all of the three sites, and as shown here, the voltages were different. In the sites 1 and 2, the saturation voltages were −2.0 V and −2.2 V, respectively. However, in the site 3, the saturation voltage was −4.2 V. In the case in which more negative bias voltages were applied beyond the saturation voltages, the PL intensity did not return, even when the voltage was returned to the initial voltage (See Supplementary Fig. S3). It is known that MoS_2_ crystals become unstable when holes are injected into them^22^. Therefore, we assume that the saturation voltages would be charge neutral points. If the large amount of n-dopants at 0 V induced the decrease in the PL intensity^19^,^20^,^21^, and thus, the amount of initial n-dopants would be more than that at the brighter site. A schematic model is shown in Fig. 2c. Thus the charge neutral points in the darker site were more negative than those in the brighter sites. In the case of sites 1 and 2, site 2 was darker than site 1, and the saturation voltage of site 2 was more negative than site 1. This behavior is consistent with the model shown in Fig. 2(c). However, in the case of site 3, the PL intensities were more than that of site 1 and 2 at 0 V, however, the saturation voltage of site 3 was more negative than that of site 1 and 2. To understand this phenomenon, it is necessary to assume the difference in the PL quantum efficiency, as shown in the model of Fig. 2d. It has been known that unsaturated Mo/S bonds^23^ and sulfur vacancies^24^ act as non-radiative recombination centres for excitons and influence the quantum yield. Those results indicate that initial doping level and PL quantum yield significantly influence on the PL intensities. The observed differences in the saturation voltage points and the maximum PL intensities indicate that both the initial doping level and PL quantum yield are not homogeneous in a single MoS_2_ monolayer sample.

### Near-field assisted nano-fabrication of monolayer MoS_2_ crystals in the negatively biased region

**Figure 3:**
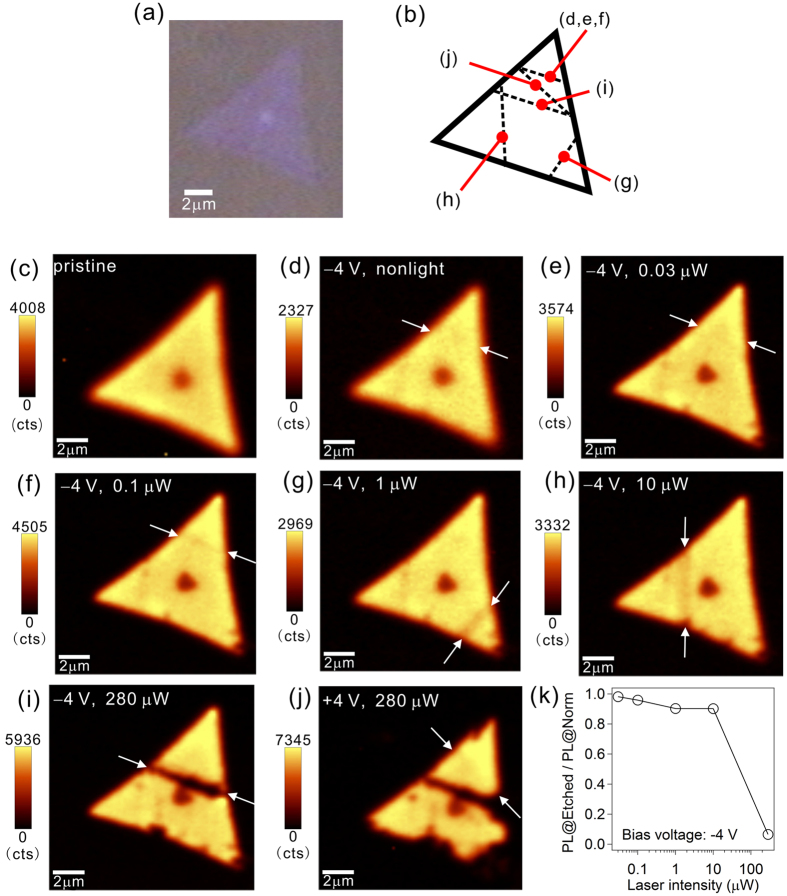
Etching of the MoS_2_ monolayers using near-field illumination. (**a**) The optical microscope image of a selected monolayer of MoS_2_ before near-field etching. (**b**) Line-scan traces performed in the panels (**d**–**j**). (**c**) The confocal PL intensity mapping image before the line scans. (**d**–**j**) Confocal PL intensity mapping images after each line scan. The bias voltages and laser intensities, which were introduced to the cantilever, during the line scans are shown. (**k**) Relationship between the PL intensity at the etched sites divided by that at the normal sites (PL@Etched/PL@Norm) and the introduced laser light intensity.

By using the phenomena of the structure instability of the crystals in the negatively biased regions described above, we etched MoS_2_ crystals using near-field techniques in combination with DC bias voltage. In Fig. 3, we demonstrate how the MoS_2_ could be etched by illumination using near-field in combination with an applied negative bias voltage. First, we conducted SNOM cantilever line scans without near-field light. At −4 V, the crystal was not etched by the line scan (Fig. 3d); thus with this negative voltage, the crystal was not decomposed (Fig. 3d). However, we found that the crystal was etched by irradiation of near-field light at this negative voltage. Figure 3e–i indicate how the crystal was etched by illumination using near-field light at this negatively biased voltage. We performed line scans, which are indicated by the white arrows and shown in Fig. 3b, at −4 V under near-field illumination with a series of laser-light intensities. As demonstrated previously, at −4 V without illumination from near-field light, the crystal was not etched; however, when we increased the intensity of the near-field light, the crystal was etched. As shown here, using laser light with an intensity of 280 μW, which was the intensity before entering the cantilever, the etched line was clearly observed after the line scan. Fig. 3k shows the relationships between the etching capability and the intensity of the laser light, indicating that near-field light was crucial to achieve etching. When the bias voltage was oppositely set as + 4 V, the MoS_2_ crystal was not etched, even when the light intensity was 280 μW (Fig. 3j). Thus, application of DC negative bias voltage was also crucial. This result, no etching using a positive bias voltage under near-field illumination, suggests that the observed etching mechanisms were neither thermal heating due to near-field illumination nor mechanical scratches due to cantilever contact. We assume that unique photochemical reactions, such as photooxidation^25^, could be one of the possible origins for the observed etching of MoS_2_ crystals by near-field illumination in combination with the applied negative bias voltages.

**Figure 4:**
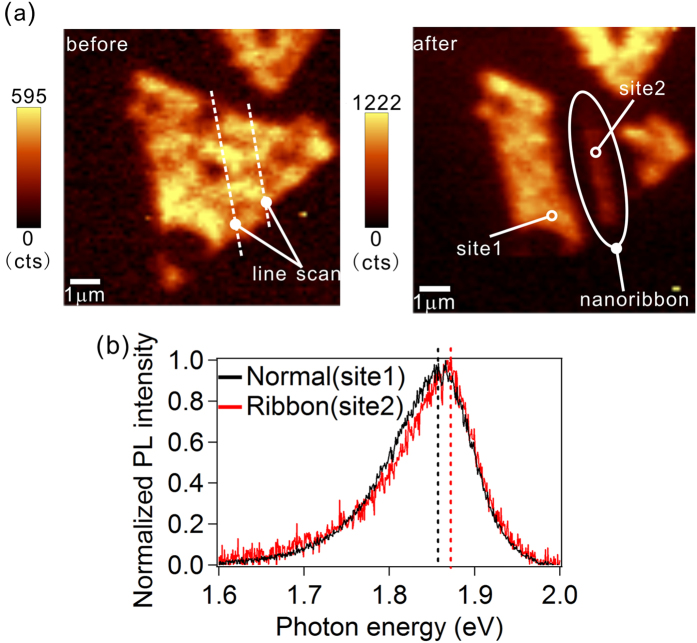
Fabrication of a MoS_2_ nanoribbon using near-field illumination. (**a**) Local PL mapping images before (left) and after (right) fabrication of the MoS_2_ nanoribbon by using near-field illumination under a negative bias voltage. The bias voltage during the line scans was set as −7 V. (**b**) Local PL spectra on a normal site (site 1, black) and on a site in the nanoribbon (site 2, red) shown in panel (**a**).

After achieving the above etching results using our electric field SNOM techniques, we tried to fabricate nanoribbon structures with TMDCs. Developing a technique to shape TMDC monolayer crystals into a nanoribbon shape is an important step toward realizing integrated circuits using their novel semiconducting properties^26^. Figure 4 shows our artificial MoS_2_ nanoribbon, which was etched from a triangular structure of a MoS_2_ monolayer crystal. The width of the nanoribbon was approximately 500 nm. We observed a slight blueshift (approximately 20 meV) for the PL peak after the formation of the nanoribbons (Fig. 4b). This blueshift was caused by a screening effect on the exciton binding energy due to the increase of the electron densities at the edge sections because such screening is known to extend to a distance of approximately 500 nm^27^. As demonstrated here, by using our electric field SNOM techniques, we fabricated nanoribbons with a width that could influence the optical properties of TMDC monolayers.

In this study, we developed an electric field-effect scanning near-field optical microscopy system that can reveal local optical properties and simultaneously tune optical properties due to an electric field applied from the cantilever. We revealed reversibly modulated local PL signals by shifting the bias voltage, suggesting electron injection at local sites on the nanometre scale. We observed the site dependence of the PL saturation bias voltages in the negative regions. Differences of the maximum PL intensities and the saturation voltage points between sites suggested that the positions of the charge neutral points as well as quantum light emission efficiencies are inhomogeneous on a single monolayer crystal. We also found that photochemical reactions were induced by near-field illumination under negative bias voltages that could etch the crystal. By using etching characteristics, we fabricated a MoS_2_ nanoribbon from a triangular structure of a monolayer crystal by near-field illumination.

Near-field light is the only way to manipulate light at the nanoscale, with which one can overcome the limitation by the diffraction of light. However, its very low light intensity, typically in the order of 10 nW μm^−2^, presents a major technical challenge, and accordingly hardly any one has expected near-field light can be applied to etching of TMDCs. Laser thinning and patterning of MoS_2_ have been reported^25^,^28^,^29^. However, a laser power of several 10 μW μm^−2^ is required for them, which is by an order of 3 higher than the maximum available intensity of near-field light, several 10 nW μm^−2^. For that reason, near-field light techniques, if ever, have been used just as an analysis tool to diagnose the local optical properties of TMDCs. In contrast, we revealed that we can etch MoS_2_ with near-field light by applying an additional DC bias voltage from the cantilever. In conventional laser etching techniques, there is a limit how thin the etching region can be fabricated because of the diffraction limit of light. We demonstrate we can break through the limit using near-field light. Indeed, we achieved the etching width of 200 nm with visible near-field light (Supplemental Fig. S5). We believe that it is a very positive indication of a breakthrough in the application of near-field light microscopy technique from a mere analysis tool to a practical manipulation tool to tune local structures and properties of TMDCs in nano-scale.

## Methods

### MoS_2_ synthesis

Monolayer MoS_2_ crystals were synthesized according to a previous report^30^. MoO_3_ powder (Aldrich, 99.98% purity, 20 mg) was used as a precursor. The raw material was put into an alumina boat. Then a SiO_2_/Si substrate (300 nm thick SiO_2_ layer) was placed on top of the boat in front of the MoO_3_ powder. The materials were placed into a 3 cm diameter quartz tube surrounded by an electric furnace. First, the raw material was subjected to an oxygen reduction process supplied with Ar/H_2_ gas at a flow rate of 1 cm^3^ min^−1^. Once the temperature of the material reached 500 °C, the temperature was maintained for 30 min. Then, the Ar/H_2_ gas flow was stopped and heated to 900 °C with N_2_ gas flow at a flow rate of 100 cm^3^ min^−1^. Once the MoO_3_ was heated to 800 °C, a sulfur flake (Wako, 99.999% purity, 200 mg) was heated to 400 °C. Then, after the temperature of the MoO_3_ reached to the set point (900 °C), the temperature was maintained at 900 °C for 10 min with the same amount of N_2_ flow. Then, after 10 min of heating, both the MoO_3_ and sulfur were immediately cooled to room temperature by an electric fan.

### Sample preparation

The SiO_2_/Si substrate on which the MoS_2_ crystals were synthesized was spin coated with PMMA (concentration of 5% relative to the chloroform solvent). Then, the substrate was dipped into a 2 mol L^−1^ KOH aqueous solution until the PMMA film peeled away. Then, the peeled PMMA film containing MoS_2_ crystals was transferred onto an ITO substrate. Finally, the substrate was refluxed by using chloroform and ethanol (each three times) and annealed for 60 min at 200 °C in a 10^−4^ Pa vacuum to drive away contaminants on the sample.

### SNOM PL spectroscopy measurements

The local PL measurements were performed by using a commercial SNOM system (WITec, alpha300RAS). The SNOM cantilever tip and cantilever metal base were electrically connected by Ag paste. Then, the cantilever metal base was connected to an electric circuit by magnets, after etching its surface to remove the oxide layer. SNOM PL measurements were performed using contact mode. The local PL was excited by evanescent light generated from a single wave laser (532 nm green laser) through the cantilever with an aperture of approximately 60 nm (incidence lens: ZEISS EC EPIPLAN, 20 × , NA: 0.4, WD: 3.0 mm). The transmitted PL signals were detected through an objective lens (Nikon MRH08630, 60 × , NA: 0.7, WD: 2.62−1.8 mm), located underneath the sample, using the appropriate integration time and accumulation to reduce the noise signal. Then, the PL signals were analysed using a monochromator (WITec, UHTS 300) and charge-coupled device (Andor, DV401A−BV−352).

## Additional Information

**How to cite this article**: Nozaki, J. *et al*. Manipulation of local optical properties and structures in molybdenum-disulfide monolayers using electric field-assisted near-field techniques. *Sci. Rep.* **7**, 46004; doi: 10.1038/srep46004 (2017).

**Publisher's note:** Springer Nature remains neutral with regard to jurisdictional claims in published maps and institutional affiliations.

## Supplementary Material

Supplementary Information
