# Supplementary material for: Manipulation of local optical properties and structures in molybdenum-disulfide monolayers using electric field-assisted near-field techniques
Source: Sci Rep. 2017 Apr 5;7:46004. doi: 10.1038/srep46004 (PMC5380953; doi:10.1038/srep46004)
Supplement: Supplementary Information [file srep46004-s1.pdf]

Supplementary Information

Manipulation of local optical properties and structures in molybdenum-disulfide monolayers using electric field-assisted near-field techniques

Junji Nozaki, Musashi Fukumura, Takaaki Aoki, Yutaka Maniwa, Yohei Yomogida, and Kazuhiro Yanagi*

Department of Physics, Tokyo Metropolitan University, Hachioji, Tokyo 192-0397,Japan

*E-mail: yanagi-kazuhiro@tmu.ac.jp

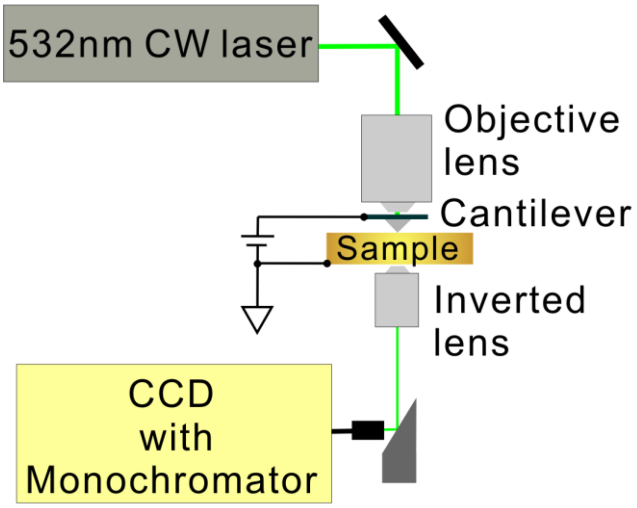

Supplementary Figure S1. A schematic illustration of our electric-field assisted scanning near-field optical microscopy setup. For measurements of transmission images described in this supplementary information (Figs. S5 and S6), the 532 nm CW laser was replaced to a supercontinuum light source (WhiteLase micro, Fianium).

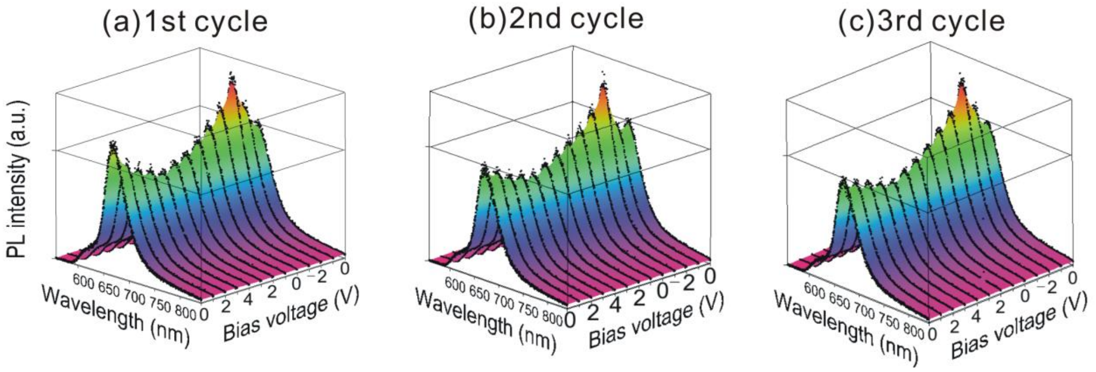

Supplementary Figure S2. Photoluminescence modulations by electron injection at a selected local site of MoS_2 monolayer crystal.

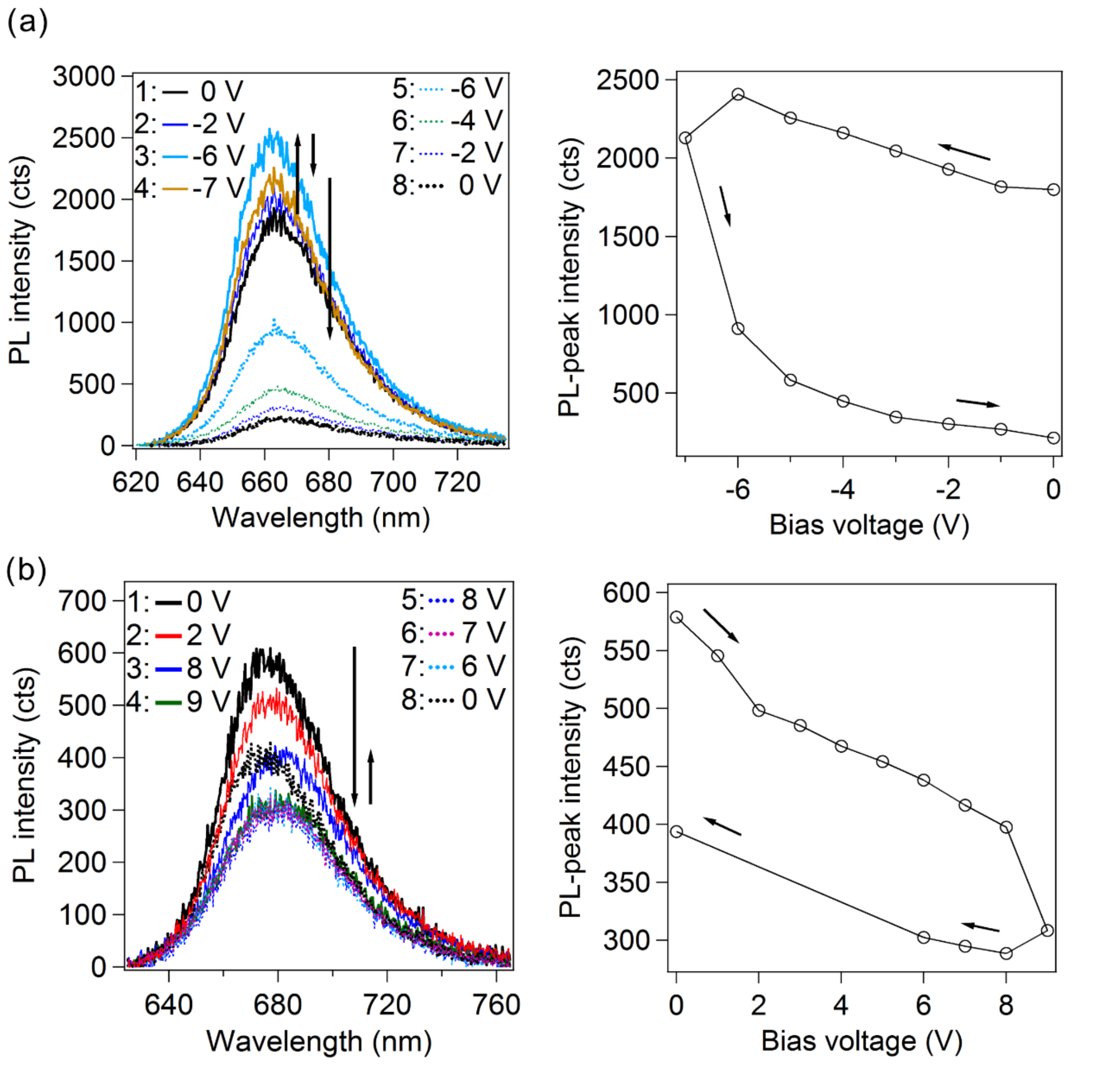

Supplementary Figure S3. (a) Photoluminescence modulations at a site of a MoS_2 monolayer crystal by negatively biased voltage beyond a voltage range that did not degrade the crystal. (b) Photoluminescence modulations at a local site of a MoS_2 monolayer crystal by positively biased voltage. When we shift the voltage more beyond the range described in the main text, as shown here, we observed significant hysteresis of the PL intensities as the shift of the bias voltage. The slight shift of PL peaks by the shift of the bias voltage would be caused by the increase of trion by doping as reported by Ref. 7 in our main text.

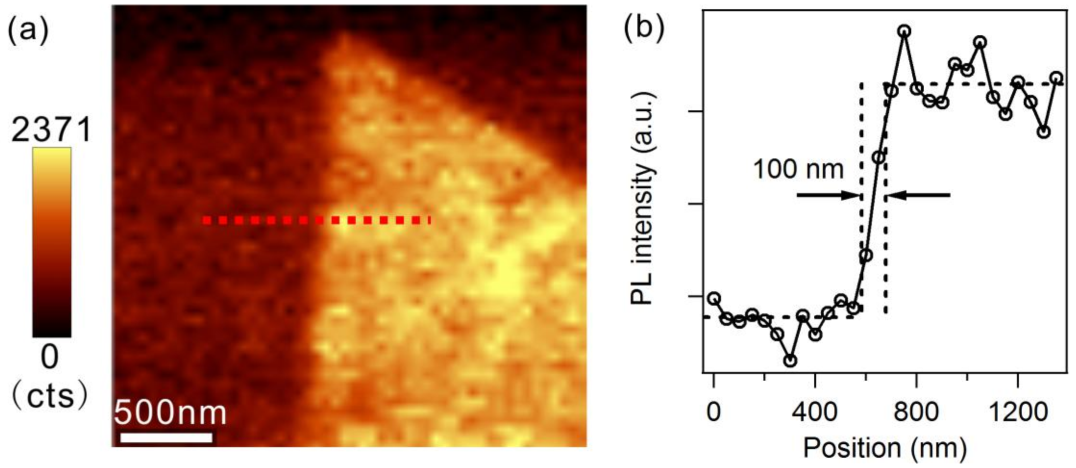

Supplementary Figure S4. (a) A near-field PL image of an apex region of a monolayer MoS_2. (b) A PL intensity profile across the red dotted line shown in the panel (a). This profile shows that the spatial resolution of our SNOM system is approximately 100 nm.

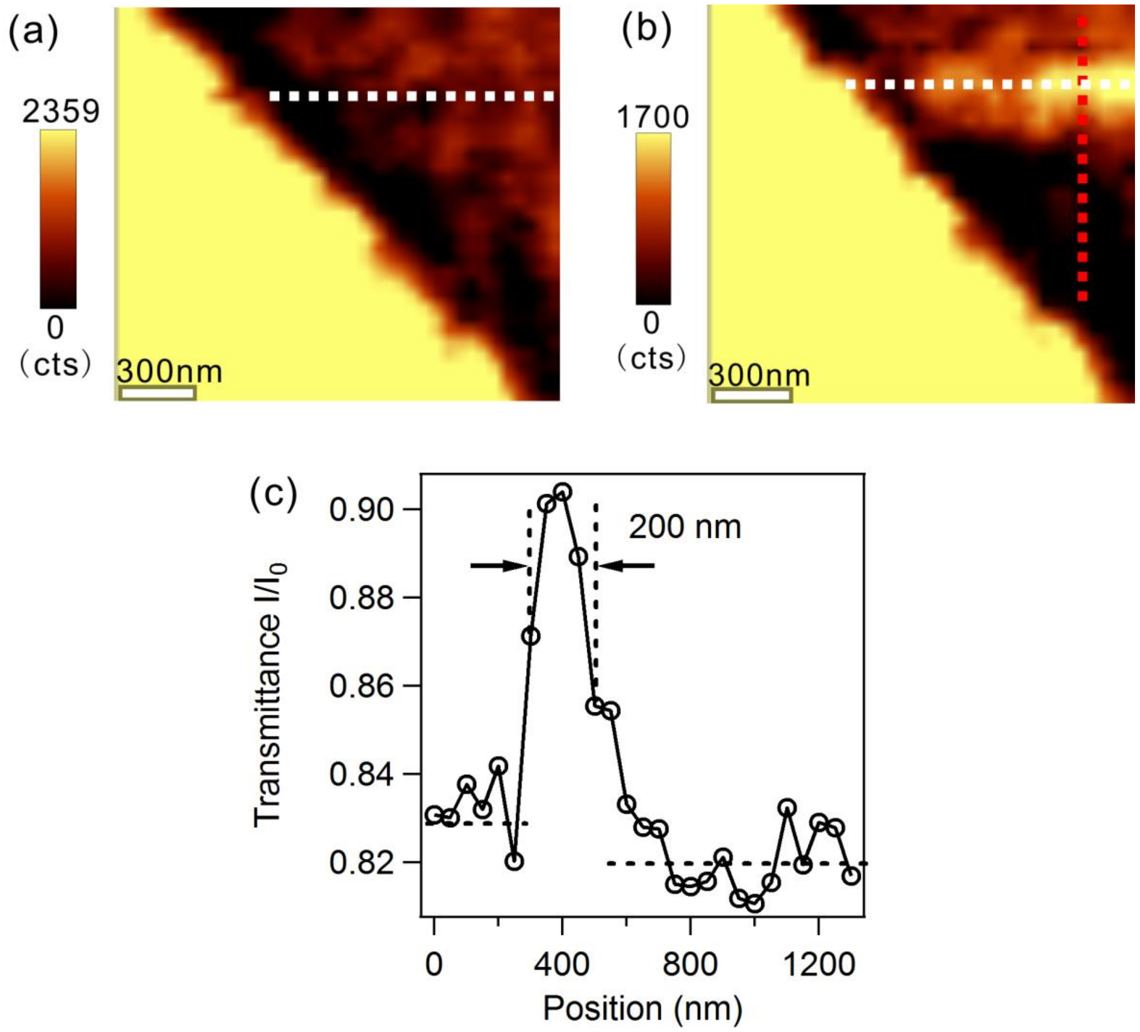

Supplementary Figure S5. Near-field absorption images using 460 nm light (WhiteLase Micro supercontinuum laser, Fianium) before (a) and after (b) near-field assisted etching of a MoS_2 monolayer crystal. The etching was performed along a white dotted line shown in the images. (c) A transmittance line profile along a dotted red line shown in the panel (b). This profile indicates the etching width of 200 nm.

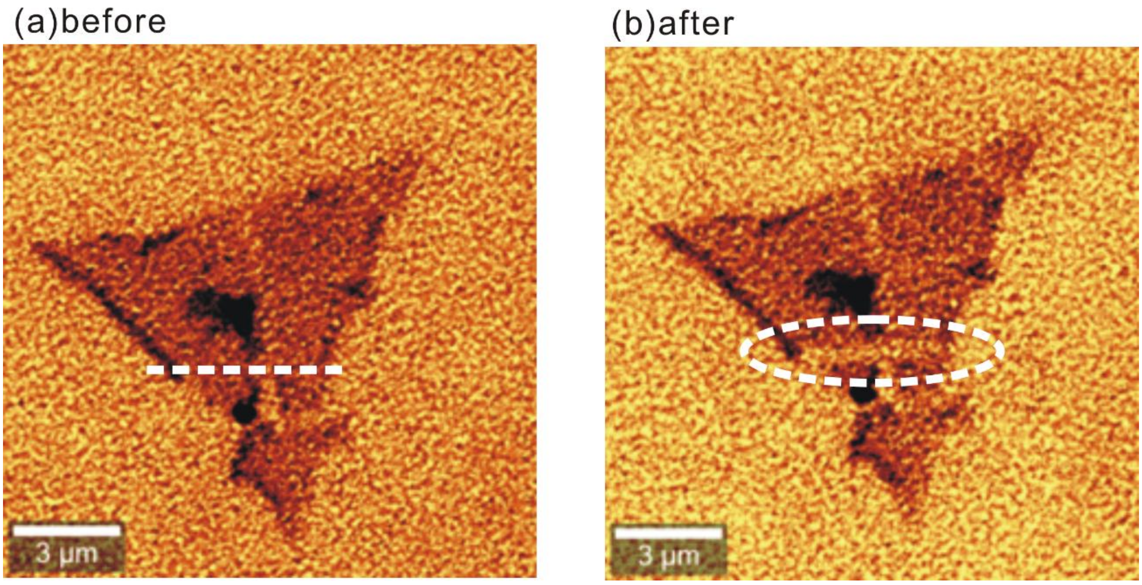

Supplementary Figure S6. Near-field absorption images using 460 nm light before (a) and after (b) etching of a MoS_2 monolayer crystal. The pictures clearly indicate the decomposition of the MoS_2 monolayer in the etched part of the crystal.

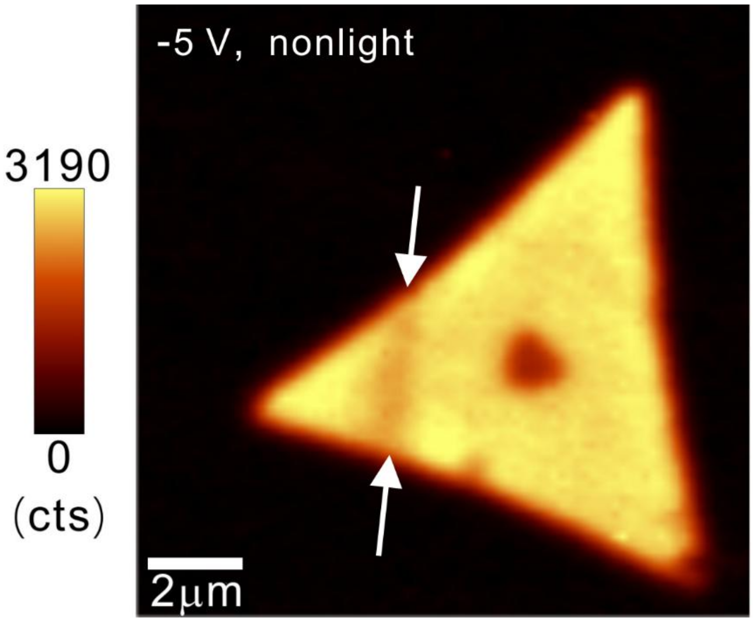

Supplementary Figure S7. As described above, a large negative bias voltage sometimes decomposes the crystal. Thus if we perform a line-scan at a large negative bias voltage, a crystal was sometimes etched. This figure indicates that a confocal PL mapping image of MoS_2 monolayer crystal after line scan at -5 V without near-field light. Line scan was performed along a line between the white arrows. The figure suggests the crystal was slightly etched by a line-scan at this negative bias voltage.
